# Supplementary material for: Decrease in All-Cause 30-Day Mortality after Bacteraemia over a 15-Year Period: A Population-Based Cohort Study in Denmark in 2000–2014
Source: Int J Environ Res Public Health. 2021 Jun 2;18(11):5982. doi: 10.3390/ijerph18115982 (PMC8199663; doi:10.3390/ijerph18115982)
Supplement: Supplementary file 1 [file ijerph-18-05982-s001.zip › ijerph-1186046-supplementary.pdf]

International Journal of
Environmental Research and Public Health

MDPI

Supplementary Materials

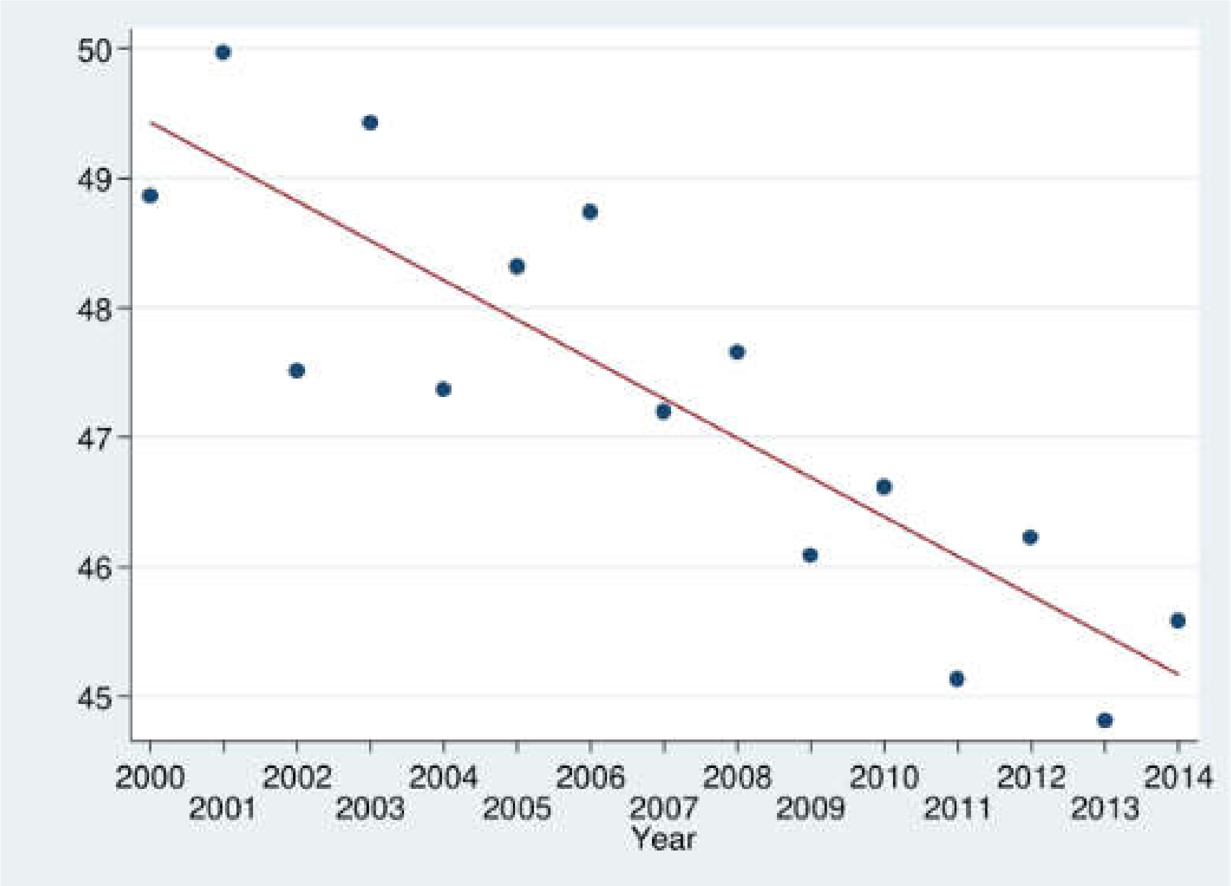

Figure S1. % females among first-time bacteraemia episodes, by year, with a trend line.

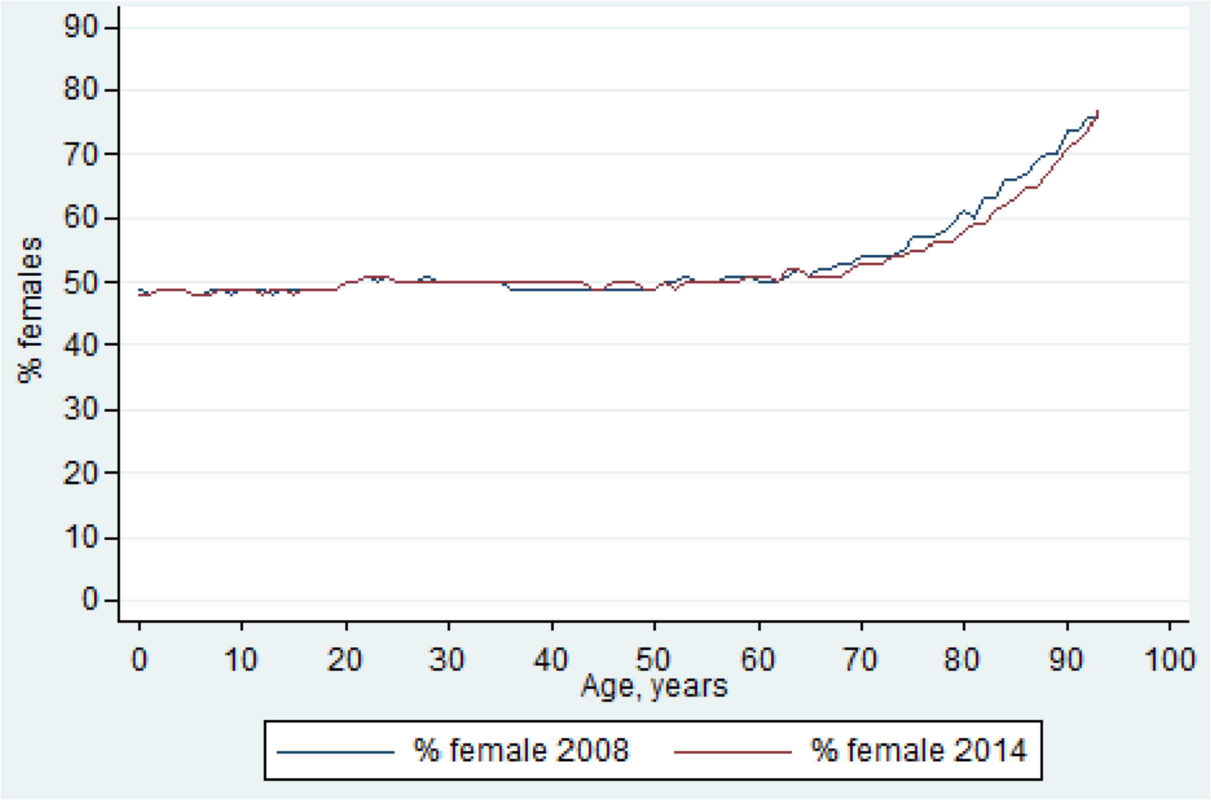

Figure S2. Female sex distribution in background population.
